# Implementation of the self-sufficiency matrix (SSM) to support diagnosing people with complex social needs at the Social Services of Catalonia

**DOI:** 10.1192/j.eurpsy.2024.123

**Published:** 2024-08-27

**Authors:** C. Salrach

**Affiliations:** ^1^Avedis Donabedian Research Institute, Barcelona; ^2^Universitat Autònoma de Barcelona (UAB), Sant Cugat del Vallès; ^3^Network for Research on Chronicity, Primary Care, and Health Promotion (RICAPPS), Barcelona, Spain

## Abstract

**Abstract:**

The Department of Social Rights of the Generalitat de Catalunya considered using a unique tool to identify people with complex social needs at Social Services centres and to support the diagnosis processes. After conducting a thorough search and selection process for various tools, the self-sufficiency matrix (SSM), a Dutch tool, was ultimately chosen.

The tool was adapted to the Catalan context through a transcultural translation process, which included a pilot and validation process. This resulted in the creation of the Catalan matrix (SSM-CAT).

A comprehensive implementation program was defined to start the adoption of the tool at basic social services. This program included training trainers and providing online training with practical cases. The implementation process was accompanied by support and monitoring to ensure success.

Through this process, over 3,468 professionals (including 334 trainers) received training on the self-sufficiency matrix, and 31,354 individuals who received basic social services in Catalonia were evaluated. In Barcelona, a more thorough monitoring of the implementation was conducted, assessing a representative sample of the care provided (6,916 individuals attended) generating a more accurate description of the situation of the people attended by social services in the city of Barcelona.

**Disclosure of Interest:**

None Declared

